# Tau regulates the localization and function of End Binding proteins in neuronal cells

**DOI:** 10.1186/2193-1801-4-S1-L16

**Published:** 2015-06-12

**Authors:** Carmen Laura Sayas, Elena Tortosa, Flavia Bollati, Sacnicte Ramírez-Ríos, Isabelle Arnal, Jesús Avila

**Affiliations:** Centre for Biomedical Research of the Canary Islands (CIBICAN), Spain; Institute for Biomedical Technologies (ITB), Italy; University of La Laguna (ULL), Spain

**Keywords:** Tau, end binding proteins, microtubule dynamics

Tau is a classical microtubule-associated protein known to regulate microtubule stability in neurons. In our study, we have addressed the putative crosstalk between tau and End binding proteins 1 and 3 (EB1/3), the core microtubule plus-end tracking proteins (+TIPs), in differentiating neuronal cells. We show that tau and EB proteins interact directly and that the cellular distribution and mobility of EB proteins depends on tau localization and expression levels. Moreover, our data reveal that tau is essential for the proper localization of EB1 at the medial-distal region of the axon shaft in developing neurons. In summary, we provide evidence for a new function of tau protein as a direct regulator of EB1/3 proteins. This further indicates that the interplay between classical MAPs and core +TIPs may be important for the fine-tuned regulation of microtubule dynamics and stability during neuronal differentiation.

